# Defibulation can recall the trauma of female genital mutilation/cutting: a case report

**DOI:** 10.1186/s13256-022-03445-0

**Published:** 2022-05-24

**Authors:** Gianmarco Taraschi, Emily Manin, Francesco Bianchi De Micheli, Jasmine Abdulcadir

**Affiliations:** 1 Department of Pediatrics, Obstetrics and Gynecology, Geneva University Hospitals, 30 Bld de la Cluse, 1211 Geneva, Switzerland; 2 Weill Cornell Medicine, 445 E 69th St, New York, NY 10021 USA; 3 Faculty of Medicine, Geneva University, Geneva University Hospital, 1211 Geneva 14, Switzerland

**Keywords:** FGM/C PTSD, Defibulation, Pregnancy

## Abstract

**Background:**

Women with female genital mutilation/cutting are known to have psychological sequelae from the cutting and other traumatic experiences. However, very few studies report immediate psychological sequelae of genital reconstructive surgery in this population. The present case is the first to our knowledge to report post-traumatic stress disorder symptoms immediately following defibulation, a procedure common in women with female genital mutilation/cutting type III.

**Case presentation:**

We present the case of a 31-year-old Sudanese nulliparous woman in the second trimester of pregnancy with female genital mutilation/cutting type IIIa who was referred for antepartum defibulation to facilitate a vaginal birth. Immediately after an uncomplicated surgery under local anesthesia and just before the first micturition, she developed post-traumatic stress disorder symptoms and suddenly recalled the traumatic experience of her first micturition after female genital mutilation/cutting when she was a child in Sudan. The woman was offered psychiatric follow-up with psychotherapy for 4 months and a short course of benzodiazepines. She had fully recovered by the time of delivery, 4 months after surgery.

**Conclusions:**

We discuss the possibility of recall of a past traumatic experience of female genital mutilation/cutting during defibulation or other genital surgeries. We review the benefits and risks of defibulation, the impact of this procedure, and the setting and timing in which it is performed, focusing on women’s mental health and psychological support.

## Background

Defibulation is a surgical procedure to expose the vaginal introitus and urethral meatus in women living with type III FGM/C [1]. During the procedure, the surgeon incises the midline scar tissue, uncovering the vaginal orifice, the external urethral meatus, and eventually the clitoris, if uncut. The cut edges are then sutured to reconstruct the labia. Defibulation is a technically simple operation that can be performed under local, locoregional, or general anesthesia, with very uncommon physical complications such as anesthesia-related complications, bleeding, injury to nearby organs (for example, urethra or clitoris), infection, pain, and spontaneous adhesion of the labia [1]. Defibulation is an intricate cultural, body image, and physiological change [2], and some women may be reluctant or refuse the procedure if not informed and counseled [3]. Women may fear dissent from their cultural or religious community or their husband [2] or believe that “closed genitalia” is a mark of virginal status [6], essential for male pleasure, or simply “normal.” Conversely, Somali men report distress regarding sexual intercourse with cut women because of the physical and psychological pain they inflict on their partner [4]. Finally, women with FGM/C type III may find a small vaginal opening beautiful and feminine, thus defibulation has the potential to lower genital self-image [5]. Our anecdotal experience has found that some patients’ genital self-image may benefit significantly from partial defibulation (up to above the level of the urethral meatus), without seriously compromising physical health. Shared decision-making should be used to decide upon the type of defibulation (partial versus total) and anesthesia (local, regional, or general) used during the defibulation procedure [1].

In this case report, we discuss a rarely studied long-term complication arising from defibulation after FGM/C, viz. PTSD exacerbation. While physical stigmata from FGM/C, particularly type III (Table 1), are well known, less information about mental and psychosexual complications can be found in literature [6, 7]. A recent systematic review and metaanalysis found a significant association between FGM/C and chronic pain score, dyspareunia, dysuria, perineal tearing, prolonged labor, and episiotomy [8] with greater severity and prevalence after FGM/C type III [6, 9, 10]. FGM/C can also lead to significant psychological trauma and mental illness. In fact, in a cohort of 66 women, 20% met the criteria for PTSD, one-third for depression, nearly one-third for an anxiety disorder, and one-sixth for all three psychopathology indicators (PTSD, anxiety, and depression) [11]. There are no studies on psychological illness and sequelae of defibulation.

**Table 1:** Female genital mutilation/cutting (FGM/C) types as classified by the World Health Organization (WHO) [12]

FGM/C type	Description
Type 1a	Removal of the prepuce/clitoral hood
Type 1b	Removal of the clitoris with the prepuce
Type 2a	Removal of the labia minora only
Type 2b	Partial or total removal of the clitoris and the labia minora
Type 2c	Partial or total removal of the clitoris, the labia minora, and the labia majora
Type 3a	Removal and appositioning of the labia minora with or without excision of the clitoris
Type 3b	Removal and appositioning of the labia majora with or without excision of the clitoris
Type 4	All other harmful procedures to the female genitalia for nonmedical purposes, for example pricking, pulling, piercing, incising, scraping, and cauterization

## Case description

We report the case of a 31-year-old Sudanese G2P0 in the second trimester of pregnancy referred to our institution to discuss defibulation. Our patient had undergone female genital mutilation/cutting (FGM/C) type IIIa as classified by the World Health Organization (WHO) [12] (“narrowing of the vaginal opening with the creation of a covering seal by cutting and apposition of the labia minora”). She stated that she was cut around the age of 5 in rural Sudan; she experienced no immediate complications. However, she suffers from long-term symptoms including superficial dyspareunia, prolonged menstrual periods with primary dysmenorrhea, and obstructed micturition with voiding efforts. Besides the FGM/C, the woman had no significant medical, psychophysical, or surgical history and was not taking any medication. She had recently resettled in Switzerland with the help of the Humanitarian Corridor. She did not experience any violence or trauma during her journey to Europe. Conversely, prior to immigrating, she experienced psychological and physical violence due to political persecution: in 2014, she was kidnapped and beaten because she had defended a group of women from a university community who had undergone a group rape. At that moment, she was pregnant at 8 gestational weeks (GW), but the pregnancy ended in miscarriage soon after this episode. She felt guilty about this loss, mainly toward her family, because of the concern she thought she had caused. She had a baccalaureat in Human Rights and had been involved in humanitarian work in her country. She was fluent in Arabic and English.

At her preoperative appointment, the woman was not depressed, although she described some symptoms of mild dysthymia (emotional instability, sleep disorders, anhedonia, and lack of appetite) mainly related to her housing situation and site of resettlement. She had two preoperative appointments before scheduling defibulation at 23 weeks of gestation: one in English and the second with a certified female Arabic interpreter. We detailed the procedure, the options for anesthesia (local and locoregional), and the option to undergo the defibulation either during her second trimester or during the first stage of labor. We discussed the anatomical changes to expect after the procedure using multimedia resources such as drawings and videos [1, 12]. In particular, we informed her that she would experience a change in micturition, which would no longer be obstructed. The patient chose defibulation under local anesthesia in her second trimester. She was instructed to apply local lidocaine cream 2 hours before the office procedure.

Defibulation was performed in our outpatient clinic under local anesthesia and was uneventful and painless. After disinfection and injection of one ampoule of local lidocaine 1%, we sectioned the tissue bridge above the urethral meatus and the vaginal introitus. The labia minora were then reconstructed with simple stitches of Vicryl 3-0. The fetus’s heartbeat was checked and found to be normal before and after the surgery.

After the intervention, the patient was dressed and ready to urinate for the first time. Going to the toilet, she quickly became distressed and burst into tears, experiencing hot flashes, tachycardia, and hyperventilation. All vital signs were regular. When questioned, she explained that she had started to experience flashbacks of the first micturition after her FGM/C when she was 5 years old. At that time, she had been afraid to urinate due to vulvar pain and had refused to drink and void her bladder. Her aunt had forced her to urinate with insults and physical violence. The gynecologist who performed the defibulation reassured and counseled the patient.

Notwithstanding her cries, urination was painless, uneventful, and psychologically relieving, even though she experienced vivid flashbacks of her childhood. The gynecologist admitted the patient to the prenatal ward to offer her psychiatric support. A few hours after the onset of symptoms, the psychiatrist’s evaluation revealed anxiety and depressive symptoms with sad thymia, anhedonia, decreased motivation, tendency to withdraw into herself, sleep disturbance, and decreased appetite, all of which had been present for several months. Besides these chronic symptoms, she also experienced an acute state of stress, with flashbacks and neurovegetative symptoms, which reactivated past traumas, not only of the FGM/C and the physical violence inflicted by her aunt but also the events she had endured in 2014.

According to the DSM 5th edition 2015 [2], the patient presented with signs and symptoms of post-traumatic stress disorder as she met the following criteria: exposure to severe violence (criterion A), unwanted upsetting memories, flashbacks, emotional distress after exposure to traumatic reminders (criterion B), avoidance of the external stimulus linked to the stressor event (micturition, criterion C), altered cognition in the form of dissociative amnesia and depression (criterion D); modification of consciousness in response to a trauma (hypervigilance, excessive startling, criterion E). Criterion F was fulfilled after a month of follow-up with persistence of flashback and anxiety surrounding micturition.

The patient was discharged 2 days postoperatively. Her psychological state was improved, presenting as mild anxiety. The psychiatrist established psychiatric and psychotherapeutic follow-up consisting of appointments every 2 weeks for 3 months, combined with lorazepam 1 mg anxiolytic treatment for the acute episode and oxazepam 15 mg for 1 month. The patient experienced a progressive decline in anxiety, depressive symptoms, and acute stress symptoms.

Postoperative follow-up was physically uneventful, and dyspareunia and obstructed micturition had completely resolved. Follow-up with the surgeon ended at 4 months after the surgery, and the patient continued to be followed by her obstetrician.

She delivered a healthy baby by vaginal delivery at 41 gestational weeks after induction for prolonged pregnancy. The delivery and post partum period were uneventful; she did not experience any relapsing symptom of anxiety, PTSD, or depression.

## Case discussion

The present report describes the case of a woman with acute PTSD exacerbation after receiving antepartum defibulation of FGM/C type III. While previous literature has explored the complex sociocultural, physiologic, and body image issues linked to the surgery [2], no studies have described acute psychological/psychiatric complications of defibulation.

However, several studies have reported on mental health in patients with FGM/C more broadly. Piroozi and colleagues found that the mean severe depression score was higher in a cohort of 122 women with FGM/C type I or II compared with 125 women without FGM/C (6.12 versus 4.60; *p* = 0.008) [13]. The link between FGM/C and psychological trauma is also acknowledged during asylum procedures [7]. The extent of psychological symptoms that ensue from FGM/C ranges from bad memories to feelings of fear, powerlessness, anger, shame, and guilt [10]. Multiple factors contribute to the extent of the psychological sequelae of FGM/C: the type of circumcision, the age at the moment of cutting, the country of origin, the level of income, the vividness of recollections, and the ability to cope [12]. Concerning the intensity of symptoms, there is some evidence that women with FGM/C type II and III present with more severe symptoms of PTSD than uncut women or women living with FGM/C type I [14]. However, for many women, FGM/C is often one of only several traumatic experiences they have undergone during their upbringing and migration [12]. Sexual and physical abuse at individual and family levels, domestic violence, and abuse during childhood are also common in this population [15]. In our case, our patient had been kidnapped and beaten for political persecution just prior to her miscarriage.

Trauma can manifest in unpredictable situations. In our case, surgery was uneventful, and it was only at the moment of inviting the patient to urinate that those bad memories came up. Providers should keep in mind this possible association of multiple traumatic experiences and address the patient’s whole life experience, rather than just an instance of trauma. However, some women are not able to openly discuss or recognize a certain traumatic experience as it is perceived as a “taboo” or normalized [16] or, like in our patient’s case, they cannot recall the trauma until a physical stimulation is applied. In our case, the patient had unconsciously removed the memories related to the cutting. Other reported coping mechanisms include taking strength and comfort from faith and religious activities or talking to friends [16]. In high-income countries, vulnerable migrant or refugee patients might struggle to seek medical advice because of social isolation, poverty, lack of recognition of the diagnosis on behalf of the patient or provider, or fear of stigmatization [17]. They may also be subjected to “iatrogenic pathologization,” a phenomenon that arises due to the broader societal rhetoric that they are “mutilated” and in need of fixing [17]. In our institution, we offer psychological support for women with history of FGM/C, particularly for patients who are planning for surgical intervention. In our patient’s particular case, psychological support was not deemed necessary as our patient did not present with any psychiatric comorbidity during the preoperative consultation. According to current WHO guidelines [6], cognitive behavioral therapy (CBT) should be considered for girls and women living with FGM/C who are experiencing anxiety, depression, or post-traumatic stress disorder (PTSD) or who will receive or have received any surgical intervention for their FGM/C.

The present case report underscores the importance of the availability of psychological support for all women living with FGM/C, particularly for those scheduled for defibulation or other surgical procedures of FGM/C, including clitoral reconstruction and treatments of scar complications as recommended by current guidelines [6]. Defibulation is a simple and generally well-tolerated procedure from a physical and psychological perspective [18, 19]. However, some women can experience acute psychological distress: in 11 years of activity of our FGM/C clinic where we perform defibulation at any moment of the woman’s life, with different types of anesthesia depending on the woman’s history, preference, and time, this is the first case of immediate postoperative psychological distress that we have been confronted with. Due to its rare occurrence, no study has reported the rate at which adverse psychological sequelae after defibulation occurs. We published the case of a young woman who experienced a relapse of PTSD caused by postoperative pain after clitoral reconstruction [20]. Multidisciplinary consultations with psychiatric and/or psychological care should be available and accessible. In low- and middle-income settings, where multidisciplinary consultation is seldom available, other forms of assistance are suitable: the WHO recommends interventions that can be safely delivered by community health workers and the use of anonymous internet-based self-help tools for management of patients with mild PTSD [21].

Risk factors for psychological sequelae should be screened; however, in our case, the patient did not recall her traumatic experience during the preoperative consultation. As in Knipscheer (2015), “the underreporting of symptoms could also be owing to different perceptions (not the circumcision but other stressors would be responsible for the current complaints/symptoms) or taboos (being ashamed to talk about the problems, feeling a sense of stigma)” [12]. Furthermore, all obstetrician/gynecologists, family medicine practitioners, midwives, and nurses should be educated on the risk of retraumatization during antepartum and intrapartum defibulation.

## Conclusions

Defibulation for women with FGM/C type III is a safe surgery to treat genitourinary and sexual complications, allow physiologic delivery and sexual intercourse, and facilitate gynecologic and obstetric procedures. Previous literature has explored the complex sociocultural, physiologic, and body image issues linked to the surgery [2]. This is the first case report that presents and discusses its possible psychological impact on the recall of trauma after surgery. Women who decide to undergo defibulation should be thoroughly informed and counseled before the procedure. Their mental health should be assessed and treated as well as their physical condition. Multidisciplinary care and follow-up with a psychiatry/psychology team before and after defibulation should be available for all women presenting with psychiatric sequelae after FGM/C.

## Data Availability

The datasets collected and/or analyzed during the current study are available from the corresponding author on reasonable request.

## Abbreviations

FGM/C: Female genital mutilation/cutting
FSFI: Female Sexual Function Index
GW: Gestational weeks
PTSD: Post-traumatic stress disorder
WHO: World Health Organization
